# A multiscale agent-based model of ductal carcinoma in situ

**DOI:** 10.1109/TBME.2019.2938485

**Published:** 2019-10-08

**Authors:** Joseph D. Butner, David Fuentes, Bulent Ozpolat, George A. Calin, Xiaobo Zhou, John Lowengrub, Vittorio Cristini, Zhihui Wang

**Affiliations:** Mathematics in Medicine Program at the Methodist Hospital Research Institute, Houston, TX 77030, USA.; Department of Imaging Physics, University of Texas MD Anderson Cancer Center; Department of Experimental Therapeutics, The University of Texas MD Anderson Cancer Center, Houston, Texas 77230, USA.; Department of Experimental Therapeutics, The University of Texas MD Anderson Cancer Center, Houston, Texas 77230, USA.; Center for Systems Medicine, School of Biomedical Bioinformatics, University of Texas Health Science Center at Houston, Texas 77030, USA; Department of Mathematics, University of California, Irvine, California 92697, USA.; Mathematics in Medicine Program at the Methodist Hospital Research Institute, Houston, TX 77030, USA.; Department of Imaging Physics, University of Texas MD Anderson Cancer Center, Houston, TX 77230, USA.; Mathematics in Medicine Program at the Methodist Hospital Research Institute, Houston, TX 77030, USA.; Department of Imaging Physics, University of Texas MD Anderson Cancer Center, Houston, TX 77230, USA.

**Keywords:** breast cancer, cell lineage, DCIS modeling, mammography, mathematical modeling, molecular signaling

## Abstract

**Objective:**

we present a multiscale agent-based model of Ductal Carcinoma in Situ (DCIS) in order to gain a detailed understanding of the cell-scale population dynamics, phenotypic distributions, and the associated interplay of important molecular signaling pathways that are involved in DCIS ductal invasion into the duct cavity (a process we refer to as duct advance rate here).

**Methods:**

DCIS is modeled mathematically through a hybridized discrete cell-scale model and a continuum molecular scale model, which are explicitly linked through a bidirectional feedback mechanism.

**Results:**

we find that duct advance rates occur in two distinct phases, characterized by an early exponential population expansion, followed by a long-term steady linear phase of population expansion, a result that is consistent with other modeling work. We further found that the rates were influenced most strongly by endocrine and paracrine signaling intensity, as well as by the effects of cell density induced quiescence within the DCIS population.

**Conclusion:**

our model analysis identified a complex interplay between phenotypic diversity that may provide a tumor adaptation mechanism to overcome proliferation limiting conditions, allowing for dynamic shifts in phenotypic populations in response to variation in molecular signaling intensity. Further, sensitivity analysis determined DCIS axial advance rates and calcification rates were most sensitive to cell cycle time variation.

**Significance:**

this model may serve as a useful tool to study the cell-scale dynamics involved in DCIS initiation and intraductal invasion, and may provide insights into promising areas of future experimental research.

## I. INTRODUCTION

REAST cancer is the most frequently diagnosed form of cancer in the United States, with 239,109 new cases diagnosed in 2014 alone (the most recent year complete statistics reported; specifically 236,968 cases in women and 2,141 in men) [1]. This translates to roughly 12% of women experiencing a positive diagnosis within their lifetime. Carcinomas in situ constitute roughly 20% of all cancers of the breast [2, 3], with the majority (~83%) occurring within the mammary gland duct, known as ductal carcinoma in situ (DCIS) [4]. DCIS is a cancer of the luminal epithelial cells, and is characterized by loss of heterogeneity of the luminal cells composing the inner layer of the mammary gland duct, resulting in dysregulated proliferation into the duct cavity. DCIS is a stage zero pre-invasive cancer (as it remains contained within the duct), and has been associated with increased risk of invasive or recurring breast cancer [2, 5]. The debate over if and when to treat DCIS continues, as even the most aggressive estimates of DCIS transitioning to invasive cancer suggest it occurs in only 1 in 3 cases [6], highlighting the need for tools to prevent overdetection and overtreatment.

**Fig. 1.**
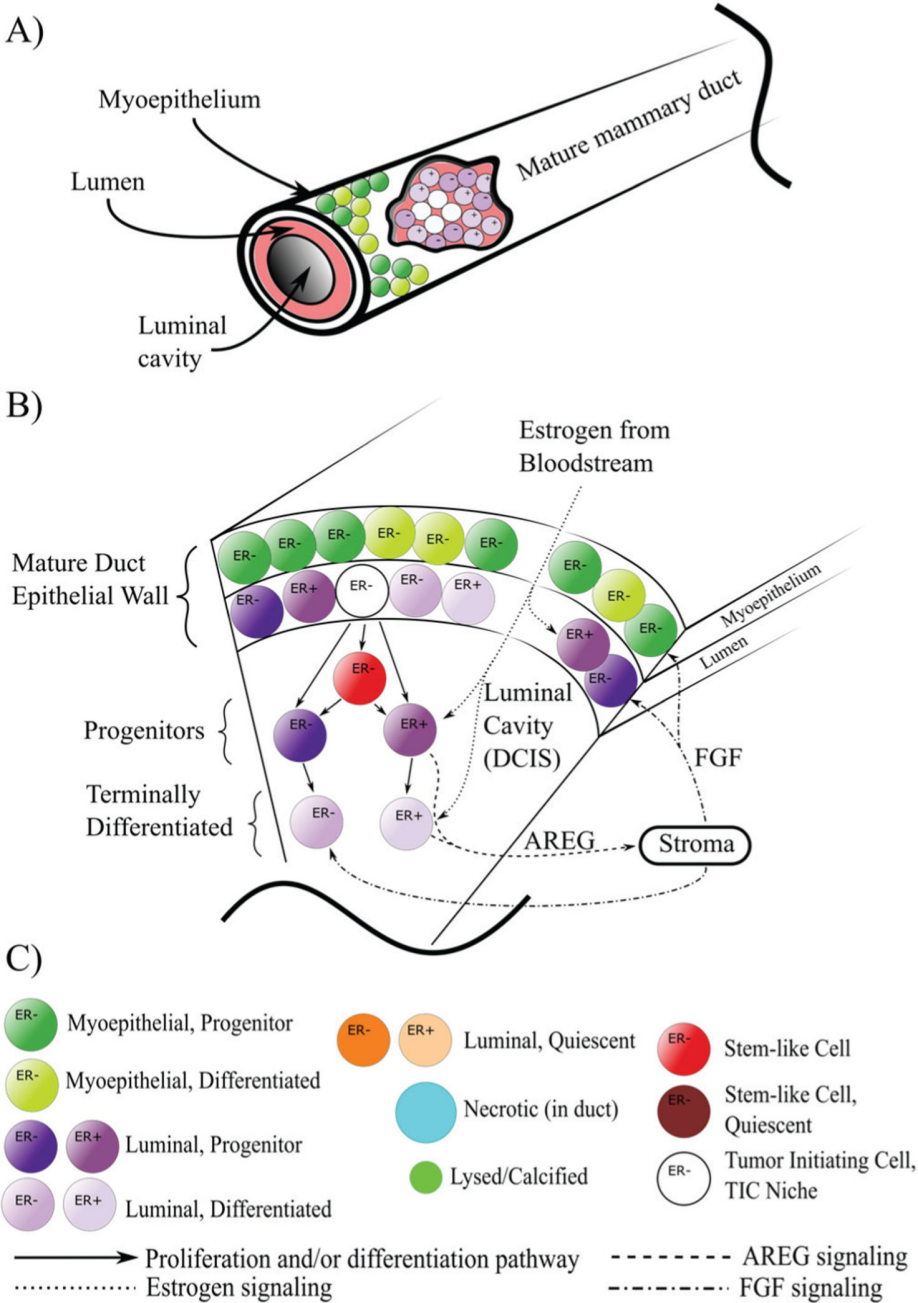
Computational domain, cell phenotype hierarchy, and signaling pathways. A) The mature mammary gland duct is composed of an outer myoepithelial layer and an inner luminal layer, both surrounding the duct cavity. At time *t* = 0, we initiate a number of tumor initiating cells (TICs) within the luminal population, initiating the onset of DCIS. B) Cross-sectional schematic of a duct section shows DCIS phenotypic hierarchy and signaling pathways. Cell signaling is as shown, with estrogen from the bloodstream signaling to the ER+ population and upregulating proliferation. These cells are stimulated to produce AREG, which leaves through the duct boundary and into the stroma, upregulating production of FGF, which reenters the duct, binding to and upregulating proliferation in the ER− phenotype; for simplicity, not all agent types are shown. C) Legend for agent color coding and signaling pathways as shown in A and B. Note that we combine all cell types into necrotic and lysed/calcified, as phenotype is no longer pertinent in these dying/dead agents. Further, all residual material from the lysis process from each individual agent (cytoplasmic contents, calcified remains, etc.) are summed together and represented by a single agent (light green).

DCIS originates from cells in the epithelial walls of the mature duct, a bilayered structure consisting of an outer myoepithelial layer and an inner luminal layer (see Fig. 1A). Both are known to be maintained by stem cell niches: small subpopulations within the gland of bipotent stem-like epithelial cells [7, 8]. Within the mammary gland, epithelial cells are phenotypically distinct based on the presence or lack of estrogen receptor α (estrogen receptor positive: ER+ phenotype) or fibroblast growth factor receptor (FGFR), and also based on stem, proliferative, or terminally differentiated phenotype. This results in distinct cellular components within the signaling pathway, where cells that express ER do not express FGF, and vice versa. Paracrine estrogen signals to ER+ cells and results in upregulation of amphiregulin (AREG) production in these cells, cascading an AREG epithelial to stromal paracrine mechanism, where AREG upregulates FGF production in the stroma. FGF then reenters the mammary epithelium and signals to the estrogen receptor α negative (ER−)/FGF receptor positive (FGFR+) cells [9]. This pathway exists within the pubertal terminal end bud during mammary gland development, and persists subsequent to gland maturity, playing an important role in mature gland maintenance (and, when abnormal, may contribute to breast cancer [10]). Although only a piece of the signaling pathway, this important cascade is critical in inducing cell proliferation involved in mammary epithelium maintenance, and is the focus of the molecular cell-cell signaling portion of this study.

As many as 70% of all breast cancers are ER+ [11]. Estrogen receptor α loss of function has been shown to be associated with transition to DCIS, which may result from an epithelial to mesenchymal transition [12]. Aberrations in the FGF receptor have also been discovered in breast cancer, and are often associated with increased FGF signaling and negative patient prognosis [10]. In addition to classification by plasma membrane receptor status, DCIS is also classified histologically, based both on cytological features and cellular architecture (cell distribution and density within the duct). The most aggressive phenotype is the comedo-type [13], which is indicated by aggressive cell proliferation and ductal advance rates, axial necrosis, and subsequent microcalcifications (small accumulations of hydroxyapatite) along the duct axis, and if the focus of our modeling study.

Hybrid agent-based modeling (ABM) [14–25] can be used as a useful tool for simulating the role of diversity in cell populations and cell-cell and cell-environment interactions, providing mechanistic interpretations of data, and making new experimentally or clinically testable predictions. In order to elucidate how disruption of the signaling pathways, cell-cell physics, and cellular phenotypic types and hierarchies involved in normal mammary gland development may contribute to DCIS, we have implemented here a hybrid, multiscale ABM of DCIS. Mathematical modeling has yielded many important insights on DCIS through both continuum [26, 27] and discrete methods [28, 29]. A number of hybrid ABMs have also been developed for studying the mechanisms of cell-scale factors and behaviors that influence DCIS progression [30, 31]. Notably, although detailed molecular signaling was not considered, Macklin *et al.* implemented a two-dimensional hybrid DCIS model, which was able to successfully predict DCIS ductal advance rates, cell density, and transition to hypoxic and necrotic states (and the resulting viable rim thickness), as validated with patient data [32–34]. Hybrid models of DCIS have also shed insights into the effects of contact inhibition, hypoxia, necrosis, calcification [35], and acidosis on DCIS architecture [29, 36], and the selective influence of these and other factors on the development and evolution of the DCIS phenotype [37].

In the present work, we seek to provide further insight into DCIS by including key signaling components, such as estrogen, AREG, and FGF pathways (Fig. 1B). We examine how phenotypic transitions within the DCIS cell population influenced DCIS progression, including the effects on phenotypic distributions and duct advance rates as limited by proliferation-dependent molecular signaling and the effects of cell density induced quiescence. By including the surrounding mature duct layer, we gain a more complete representation of cell signaling (i.e., AREG production by the ER− population within the luminal layer of the mature gland). This layer also affects estrogen and oxygen concentrations within the duct, as these molecules are used by the mature duct cells and play a key role in the epithelial to stromal signaling pathway (i.e., AREG to FGF), and has been included for future studies of myoepithelial DCIS and our ongoing work studying pubertal mammary gland development as well [38]. Both molecules cross the mature duct cell layers (and may be influenced by the appropriate cell phenotypes therein) as a fundamental part of their signaling mechanisms. Thus, we attempt to implement a more complete picture of the mammary gland environment surrounding the DCIS population through inclusion of the mature duct layers, in order to better obtain a more detailed description of the complicated interplay of molecular- and cell-scale dynamics in DCIS disease progression.

## II. METHODS

In our ABM, cells are represented as unique, discrete entities (agents), while molecular signaling profiles and molecular movement are represented as continuums through a mathematical description using partial differential equations (PDEs) which are solved numerically at each time step. A cellular phenotypic hierarchy has also been implemented based on the published literature as shown in Fig. 1B. Briefly, agents are implemented in a 3D, lattice free system, where all agent interactions and movements are determined based on cell-cell and molecular level physics. Continuum and discrete scales are explicitly linked mathematically, and feedback between the two scales is explicitly computed. At each time step, agents probe the continuum solutions for information about the molecular concentrations within their microenvironment, and then they modify these profiles based on cellular phenotype and their associated molecular production or consumption. In this work, we use the terms “consumption” to describe any molecular concentrations which are reduced by cell behavior (e.g., oxygen consumption and metabolism or the binding of signaling molecules to membrane-bound receptors), while “production” refers to all cell behaviors which increase the concentration of the molecule of interest (e.g., cells may produce proteins or they may release already-translated proteins). Agents are bound by rules which are coded to represent literature-supported cell types and behaviors, including signaling pathways, receptor expression and overexpression, cell cycle times, proliferation, and growth rates and patterns. The model was implemented in C++, with meshes generated using Trelis, mesh processing supported by Exodus II [39] and VTK [40], graphics pipeline via OpenGL, QT, and x11, FEM solutions obtained via Sundance [41], and also using various functionality from BOOST, FLANN [42], and BulletPhysics [43].

### A. Continuum Methods

**Fig. 2.**
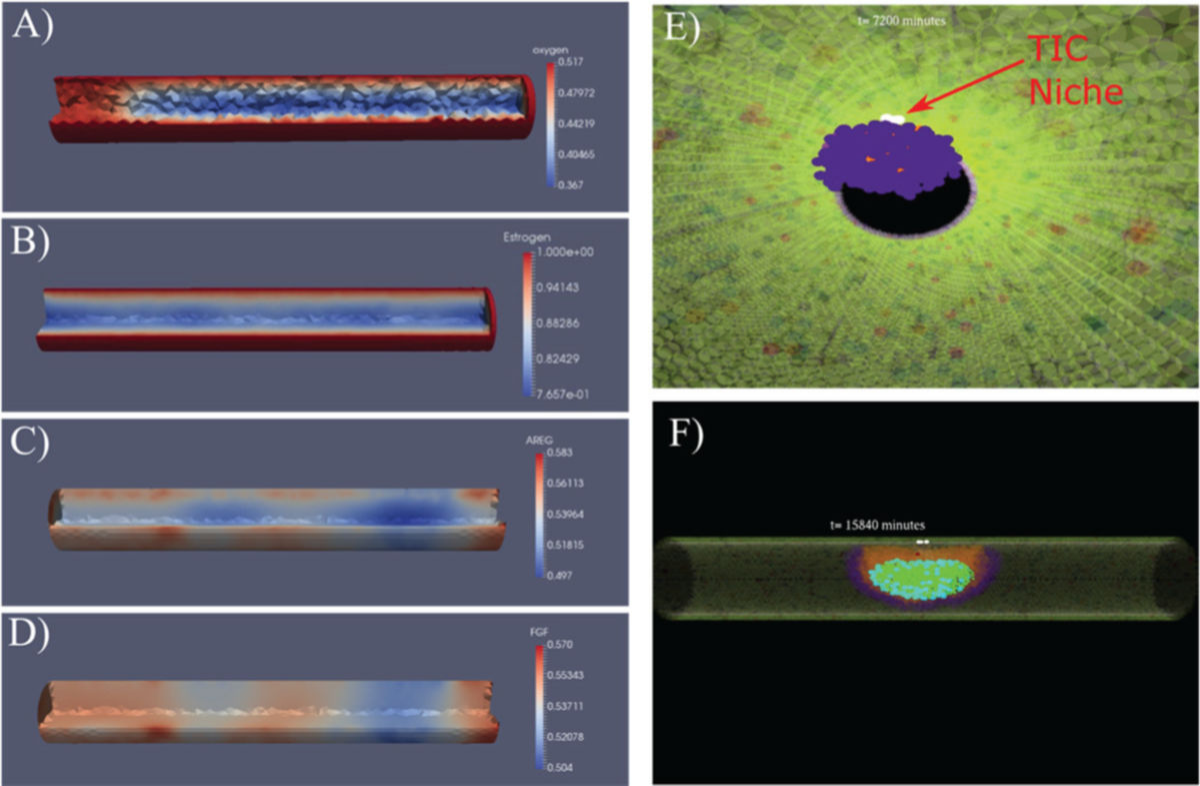
Examples of continuum (A−D) and discrete (E, F) model components. Continuum solutions from FEM within an idealized mammary gland duct are shown for A) oxygen, B) estrogen, C) AREG, and D) FGF. Oxygen enters into the duct from the boundary under Dirichlet conditions; regions shown in blue are where DCIS has reduced the local oxygen conditions. C, D) AREG is produced in the duct by ER+ cells (red: high concentrations; indicates localized AREG production) and diffuses throughout the domain and out of the duct radial boundary. The FGF boundary condition is derived from the AREG solution. E) Internal view of DCIS five days after DCIS initiation (viewpoint: inside the duct cavity looking parallel to the duct central axis); the TIC niche is shown in white (red arrow), with a growing DCIS mass (purple) seen encroaching into the duct cavity away from the TIC niche. F) An example view of DCIS 11 days after DCIS initiation; the mature duct cells and healthy DCIS progenitors are shown as transparent so the stem phenotype, as well as hypoxia and calcification internal to the DCIS may be seen clearly. A−D: 100 μm diameter duct; E, F: 200 μm diameter duct (shown for ease of visibility); all agent colors are as shown in Fig. 1C.

Molecular signaling is represented as a continuum, described mathematically using a Fick’s law description of the reaction-diffusion equation, as described in Eq. 1,

(1) $$\frac{du}{dt}=D\nabla^{2}u+R(u),$$

and based on some of our previous modeling work [38]. This PDE describes the time-dependent molecular concentration (*u*) within the computational domain (e.g., the simulated duct), as dependent on the diffusion constant (*D*), and as modified by a reaction term *R(u)*, which accounts for both molecular consumption or production *U*(*u,x*), as well as molecular degradation *L*(*u*); i.e., R(u)=U(u,x)−L(u). Time steps for continuum solutions, shorter than ABM time steps, have been tested to ensure solution stability. FEM solutions are obtained using Sundance [41], a high-level finite element method library included as part of Trilinos, a numerical methods package developed by Sandia National Laboratory on a 3D tetragonal mesh (mesh generated with Trelis meshing software; see Fig. 2A) (examples are shown in Fig. 2A–D).

Boundary conditions are defined to best describe the biological conditions for each molecule of interest. Blood is supplied to the mammary gland through a system of surrounding capillaries, which we assume are located directly on the outer duct surface and contain a rapidly replenished blood supply (through circulation) at all times. This results in a constant concentration of molecules of interest (e.g., oxygen, estrogen) at the boundary. In our model, estrogen and oxygen enter into the system numerically as constant Dirichlet boundary conditions (BCs) on the outer surface of the duct, but excluding the ends where we truncate the computational domain into a “duct section.” For each molecule of interest *m*, we apply a constant value *C*_*m*_ on all boundary nodes *x*_*b*_, as per

(2) $$u_{m}\left(x_{b}\right)=C_{m},$$

and these molecules may diffuse into the domain freely (but according to appropriate diffusion constants) according to (1). For boundary conditions, blood oxygen concentrations ($C_{O_{2}}$) is taken to be 100 mmHg [44], while female pubertal blood estrogen concentrations are lower than oxygen concentrations (median of 70.3 pMol/L [45]; and lower still post-menopause, when most DCIS occurs). These molecules may be removed by agents from the local concentration *R*(*u*) due to molecular consumption (oxygen, $\lambda_{H}\;\text{and}\;\lambda_{C}$ for healthy and cancer cells, respectively) or binding to the appropriate receptor (estrogen to ER+ agents, and FGF to ER− agents). This is summed with a molecular degradation term, *L*(*u*), which accounts for potential molecular sinks (including molecules lost to agent necrosis, lysis, and apoptosis), and the presumed uptake of molecules by other cells. These molecular losses are taken to be small, i.e., |L(u)|≪|R(u)|, but are included in the model for sake of completeness. Specifically, the total change in local molecular concentration *u* at location *x* is calculated as

(3) $$\begin{array}{l} R(x,u)=U(x,u)-L(u)\\ R(x,u)=\pm \sum_{i=1}^{n}\lambda_{i}H\left(r_{i}-\left|x-a_{i}\right|\right)u-L(u), \end{array}$$

where *r*_*i*_ and *a*_*i*_ are the radius and center of mass coordinates of the agent, respectively, *H*(*x*) is the Heavyside function, and $\lambda_{i}$ is the per-volume or per-surface area consumption or production value for the appropriate molecule (positive sign is production, and negative sign is uptake or consumption). We have made the assumption that all cells of the same phenotype (i.e., cancer vs. healthy, or as per cell receptor status, e.g., ER+/−) have the same $\lambda_{i}$ values in the work presented here.

The magnitudes of local molecular concentrations are computed as the average values from all agents with their center of mass *a*_*i*_ closest to each node (as determined through Voronoi tessellation [46], a method of subdividing the domain into regions which enclose the volume closest to each node), normalized into per-volume or per-surface area values, and applied to the appropriate node of interest numerically through application of a Dirac delta function, defined as

(4) $$U(u,x)=\frac{\sum_{i=1}^{n}\lambda_{i}u\int_{-\infty }^{\infty }\delta \left(x-a_{i}\right)\text{d}x}{n},$$

where *n* is the number of agents in the Voronoi cell. To simplify calculations, we take the total volume (and thus the total contribution to local molecular concentration) of the agent to be within the Voronoi cell containing its center of mass; thus, the total values for each cell are implemented into the continuum solution entirely at the nearest node. By subdividing the mesh into elements of similar length to the mature agent diameter, we have attempted to ensure that only a small number of agents are associated with each node, thus maintaining an acceptable degree of precision within this approximation.

AREG is sourced exclusively from cellular production by the ER+ phenotype within both mature duct layers and the DCIS population. AREG is free to diffuse out of the domain across the outer radial boundary under the same conditions that it diffuses though the domain through the implementation of homogenous Neumann boundary conditions, with molecular concentration attenuating toward equilibrium at the far-field. Biologically, the AREG that leaves the duct signals to epidermal growth factor receptor (EGFR) in the stroma, stimulating an epithelial to stromal cascade which results in FGF reentering the duct (as diagramed in Fig. 1B). Numerically, we implement this as a time-dependent Dirichlet boundary condition, defined for each time step (*t*) as a function of the per-node values of the AREG solution from the previous time step (*t* − 1) as

(5) $$u_{\text{FGF},i}(t)=u_{\text{AREG},i}(t-1),\;\left(i=0,\ldots ,n_{i}\right)$$

for each node *n*_*i*_. In this way, a direct downstream epithelial to stromal signaling mechanism is implemented, providing a reasonable approximation of the epithelial to stromal signaling pathway. An example of FEM solutions for the epithelial to stromal (i.e., AREG to FGF) pathway is shown in Fig. 2C, D. We have strived to, whenever possible, implement literature supported values in these equations. The details of how we selected, calibrated, and validated our baseline values (e.g., oxygen: $D_{\text{oxygen}\;},\lambda_{\text{H}},\lambda_{\text{C}}$; as well as for endocrine and paracrine signaling molecules estrogen, AREG, and FGF) based on extensive literature review are shown in Supplementary Materials section S1.1, and a summary of important model parameter baseline values is shown in Table I. Additionally, further details of the continuum numerical methods are provided in Supplementary Materials section S1.3.

### B. Discrete Methods

Cells in the mature mammary gland and the DCIS population are represented discretely through implementation of an ABM. Each cell agent is unique, with its own geographical coordinates, phenotype (luminal or myoepithelial, healthy or cancerous, etc.), receptor status (ER+/–), size (approximated as spheres with radius *r* to simplify the mathematics of the physics cell-cell interaction), cell generation number, and cell state. Agents proliferate as instructed through molecular signaling, and as allowed by phenotype, neighbor density, and cell cycle time restrictions. Cells in the model may only proliferate when molecular signaling thresholds are satisfied (approximated in this work via a simple binary step function), as done in our prior ABM work [38, 62–68], when they are not induced into quiescence due to agent density restrictions, and only after a full cell cycle has occurred after their last mitosis event, at a frequency *f*p, taken to be ≥ the standard cell cycle time $\tau_{p}$ = 16 hours [50, 51] (see Table I).

**Fig. 3.**
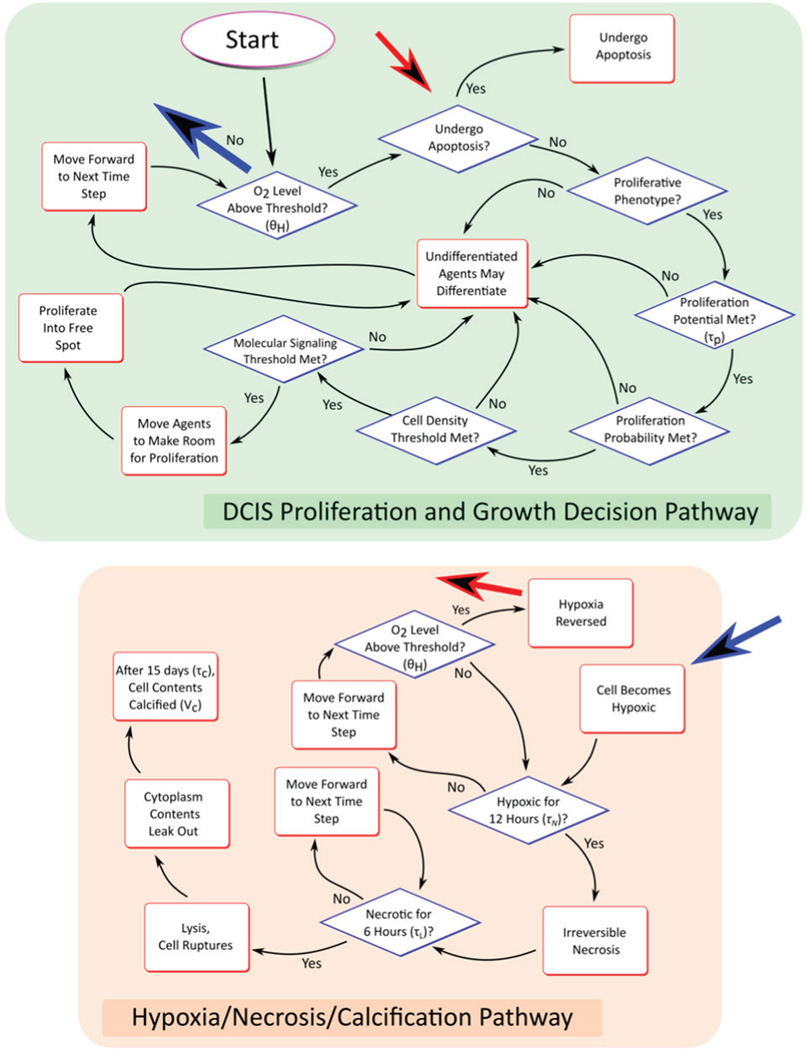
Agent decision flowchart. Agent decisions are made according to the rules shown for each time step of the discrete model. Blue and red arrows indicate transition pathways between top and bottom boxes (shown without complete arrows connecting the two regions for clarity).

A new daughter cell must complete a simulated interphase (cell growth, which is modeled explicitly by cell volume increase at each time step until the cell reaches a mature cell volume, but without explicit representation of the involved subcellular processes) before it is eligible to proliferate again. Once the last cell cycle is complete, a cell of progenitor phenotype may proliferate, provided a set of conditions are satisfied, including if relevant molecular concentrations are above a proliferation threshold and if local cell density is below a density threshold. When all conditions for the mitosis event are satisfied, the cell divides, splitting its cytoplasmic volume and plasma membrane contents evenly between its daughters. This decision-making process is described graphically in Fig. 3. All cell movement, due to both proliferation and cell-cell interactions, occurs in an off-lattice configuration, where cells may occupy any allowed coordinate within the computational domain. This is solved via a physics-engine representation of the discrete model (solved with BulletPhysics [43]), as detailed in Supplementary Materials section S2.1.

Cells follow a cell hierarchy as shown in Fig. 1B. At time *t* = 0, we allow a small number of cells in the luminal layer of the mature duct to have a tumor initiating cell (TIC) phenotype (shown in white, Fig. 1 and 2D,E). Each TIC is seeded to be at a random time within the cell cycle, through implementation of an individual counter (thus there is one unique counter for each cell) which records the time since the cell’s last mitosis event. This counter is incremented for each agent each time the ABM time is stepped forward, and the agent may not proliferate again until the counter value is at least equal to the cell cycle time $\tau_{p}$ (16 hours based on [50, 51], see Table I). TICs are taken to have unlimited proliferation potential, and may thus proliferate an unlimited number of times [69, 70], while proliferative DCIS agents are limited to a maximum number of proliferation cycles based on the literature (see Supplemental Materials section S2.2).

Aggressive cell proliferation in the absence of a properly developed vasculature (as observed in solid tumors) often results in restrictions of oxygen availability, hypoxia, and even hypoxia-induced necrosis. In our model, agents become hypoxic if the local oxygen concertation falls below a critical hypoxia threshold (e.g., $C_{O_{2}}<\theta_{H}$), and will undergo necrosis and subsequent cell lysis if the local hypoxic conditions persist longer than the critical threshold $\tau_{\text{N}}$. Quantification of these values is detailed in Supplementary Materials section S2.4, and baseline values are provided in Table I. Note that we have made the assumption that, due to mutations resultant in the cancer phenotype, apoptosis pathways are turned off in the DCIS populations; thus all cell death in the model is due to the hypoxia and necrosis pathway.

### C. Hybridization of Models

In our model, components of the continuum and discrete scales are explicitly linked mathematically. Information is directly communicated between the scales at each time step, and each scale component is directly affected by, and directly affects, the other. Agents in the discrete scale receive information about their microenvironments directly from the continuum scale. Each agent probes its microenvironment at the beginning of each time step in order to determine the local concentration of all molecules represented in the continuum scale (oxygen, estrogen, AREG, and FGF) at its location (for simplicity, agent location is taken to be its center of mass). Because the solutions of continuum molecular profiles are only known exactly at the node locations, agents must interpolate the concentration at their location from the values at its nearest nodes. Each agent identifies its nearest nodes at each time step, and agents then interpolate the value at their location from the values at the nearest nodes using linear barycentric interpolation. Agents also feedback into the continuum scale through direct modification of the continuum solutions, based in part on their phenotype (see Fig. 1B), as described in detail in Continuum Methods. Through explicit linking of discrete and continuum scales, the model is able to provide detailed information about interplay between continuum (tissue) and discrete (cell) scales, and to give useful insights into the contributions of molecular factors involved in determining behavior observed at a cellular level. Hence, the information that can be gained here with the hybrid method is not available through using either discrete or continuum methods alone.

## III. RESULTS

We have implemented our 3D DCIS model to simulate the earliest stages of DCIS in a simulated duct. The mammary ductal structure possesses significant variation in duct diameter, which shows significant variation even within a single gland [30, 71]. In a study measuring 1,285 excised human mammary ducts, Mayr *et al.* reported a mean diameter of 90 μm for the normal duct (520 samples, range 39−314 μm), but a statistically significant increased mean diameter of 314 μm (765 samples, range 60−1708 μm) in ducts with intraductal carcinoma [71]. Of the ducts measured, ~97% of healthy ducts and ~30% of ducts containing intraductal carcinoma were found to be smaller than 200 μm diameter. This significant variation in duct diameter between healthy ducts and ducts containing DCIS is due (at least in part) to mechanical stretching of the duct by DCIS expansion. Simulations presented here were performed in three different diameters of mammary gland duct sections (100, 150, and 200 μm luminal cavity diameter based on [30, 71]; i.e., the thickness of luminal and myoepithelial layers in the mature duct are not included in this measurement, but instead surround a luminal cavity of this diameter), each represented as a cylinder of duct 1 mm in length axially.

### A. Model Setup

Through initial testing (see Supplementary section S3.1), we found that the number of TICs initiated in the niche at time *t* = 0 had a negligible influence on the total DCIS extent and rate of advance for all duct sizes tested. Hence, we chose to proceed with 5 TICs assigned in the luminal layer of the mature duct at the start of all simulations. These TICs may proliferate indefinitely, placing their daughters into the luminal cavity, as determined by cell phenotypic hierarchies shown in Fig. 1. These cells may continue to proliferate, as determined by mitosis threshold rules (see Table I) and satisfaction of molecular signaling thresholds. TICs were initiated at the center of the duct axis (e.g., in a 1,000 μm axial length duct section, they would be placed as close to axial location *x* = 500 μm as possible), with all TICs in a contiguous location. For consistency, we seeded a standard set of agent locations in the mature duct layers at the start of simulations (*t* = 0; however, each non-TIC agent has a stochastically determined phenotype, making the cellular composition of each simulation unique). The DCIS population may invade the duct both radially across from the TIC niche and axially along the duct from this central locations. The total ductal axial extent is taken to be the summed magnitude of cell advance through the duct cavity bidirectionally from the TIC niche. At each time step, details of each agent (locations, phenotypes, cell states, etc.) were recorded, and results are detailed in the sections below. We note that, under the model configurations tested here, our simulation results only model the comedo-type solid DCIS architecture.

Model outputs of interest reported here include the extent of DCIS (measured as described in the previous paragraph), the associated DCIS axial advance rates (estimated as a linear best-fit to measured DCIS extent), and total DCIS cell population, as well as extent of hypoxia and calcification when appropriate. Simulation results were compared against literature-reported ductal invasion rates within the human mammary gland, and both calibration of baseline parameters and validation of successful model results were determined based on consistency between model results and literature- reported values for ductal invasion rates (reported to range from 5.5–13 mm/yr) [72, 73]. Literature-supported parameter values were used as baseline values (Table I), with perturbations applied for sensitivity analysis (i.e., Results III.D). When parameter values were not available from the literature, we calibrated the model phenomenologically to reproduce behaviors we were able to obtain from the literature (see Supplementary section S1.2). All simulations were run on the Lonestar 5 supercomputer, located at the Texas Advanced Computing Center, The University of Texas at Austin.

### B. Early-stage DCIS growth occurs in two distinct phases

**Fig. 4.**
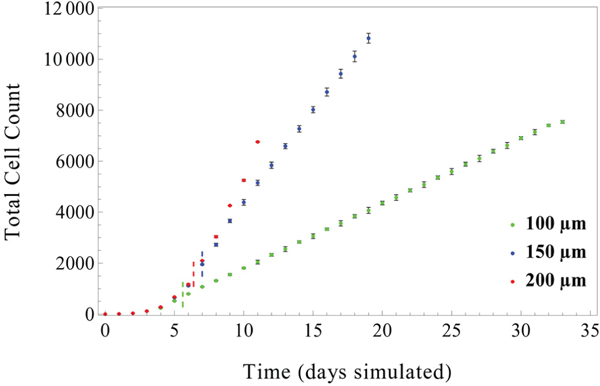
Total cell count over time for three duct sizes: 100, 150, and 200 μm internal diameter. DCIS cell population expansion occurs over two distinct phases: early exponential growth due to population doublings occurring once per cell cycle, followed by a linear growth phase (transition times between phases indicated by dashed lines, colors correspond to figure legend; found to be 5.6, 7.0, and 6.38 days in the 100, 150, and 200 μm ducts, respectively; see Table II). Subsequent to this transition, the population remains in a linear growth pattern. This effect is further accentuated in the 200 μm duct, where the total cell count increase due to disease progression is balanced by necrosis-induced cell death. All data shown with 5 TICs initiated at *t* = 0 and without signaling threshold effects. Points show mean values of 3 simulations (taken at the end of each simulated day), error bars = standard deviation.

Examination of the total cell count over time reveals two distinct phases of DCIS growth behavior: an early, transient exponential growth period, followed by an extended linear growth period, which in good agreement with previous modeling work [74–77]. This behavior is observed in all duct sizes (see Fig. 4), and for all numbers of TICs seeded in the TIC niche (data not shown). Transition between these two phases was found to occur between 5.6 to 7 days (median transition times across duplicate runs (the average of 2x runs with 5 TICs in the niche) were found to be 5.60 days, 7.00 days, and 6.38 days in the 100 μm, 150 μm, and 200 μm diameter ducts, respectively; as indicated by short dashed lines in Fig. 4). These observed exponential growth phases correspond to DCIS population doubling times of 23.3 hours, 26.28 hours, and 23.2 hours for 100, 150, and 200 μm diameter ducts, respectively. The early-stage exponential growth phase was maintained until cell-density induced quiescence reduced proliferation in the DCIS population, after which consistent linear growth rates were observed across all duct diameters. This biphasic pattern of DCIS population increase is observed to be irreversible, and once a transition from exponential to linear growth occurs, the model will remain in the linear growth pattern for the rest of the simulation. In all cases, it was observed that the rate of increase in the DCIS cell population is larger for larger duct diameters, due to a larger total non-quiescent population.

Detailed values for times to phase transition, as well as best-fit values for exponential and linear growth rates and *R*^2^ values are shown in Table II.

### C. Model parameter sensitivity analysis

**Fig. 5.**
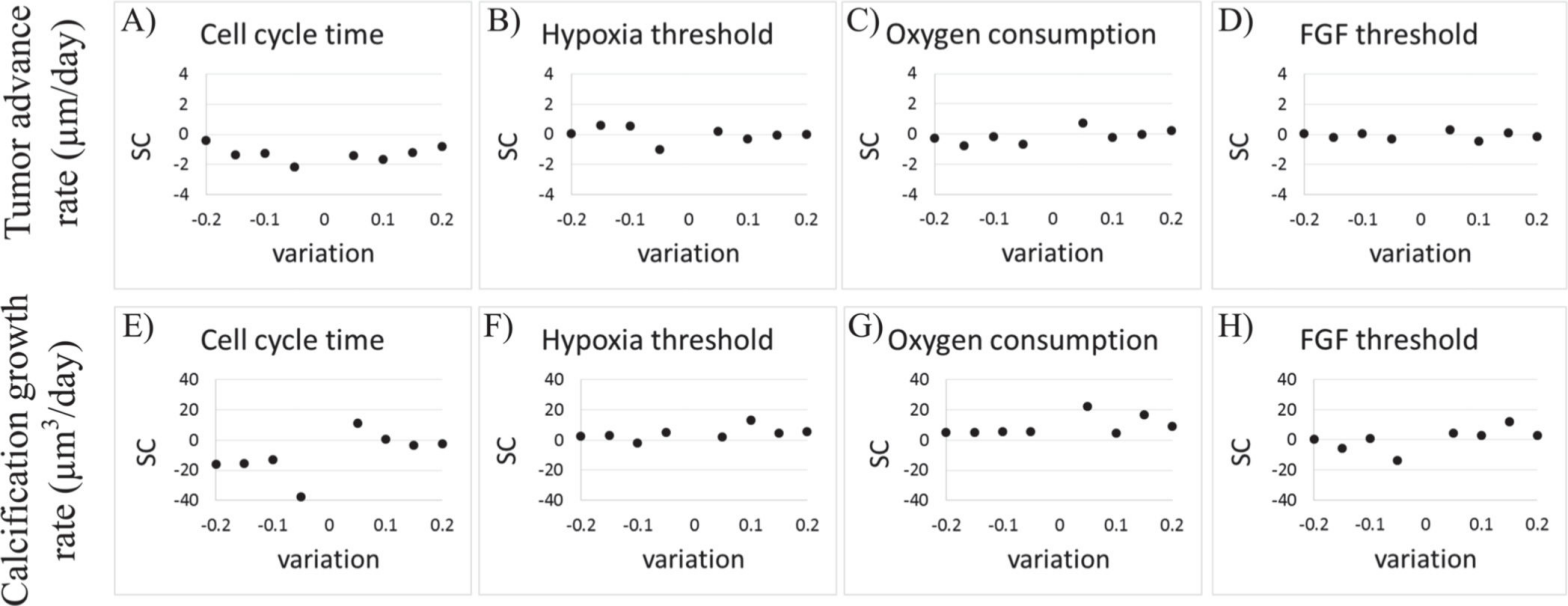
Sensitivity coefficient under parameter perturbation. We analyzed model sensitivity to perturbations in A,E) cell cycle time ($\tau_{\text{P}}$), B,F) hypoxia threshold (*θ*_H_), C,G) oxygen consumption ($\lambda_{c}$), and D,H) FGF sensitivity threshold. Model responses to parameter perturbations were quantified base on the changes in A−D) tumor axial invasion rate and E−H) calcification growth rate. Model sensitivity was calculated using a sensitivity coefficient (SC), which quantifies change in model output per change in model input parameter (see Equation (6)).

We performed a local sensitivity analysis (i.e., only one parameter is varied, while keeping all other parameters fixed at their reference values), to examine the impact of changes in parameter values on DCIS growth dynamics. In our analysis, we focused on four input parameters: DCIS cell cycle time ($\tau_{P}$), hypoxia threshold (*θ*_H_), cancer cell oxygen consumption rate ($\lambda_{C}$), and FGF proliferation threshold; and two model outputs of interest: tumor advance rate (μm/day) and calcification growth rate (μm^3^/day) in the largest duct examined in this study (200 μm diameter) over 30 days of simulated DCIS growth. These input parameters were perturbed from 0.8-fold (a 20% decrease) to 1.2-fold (a 20% increase) of the baseline values (Table I) in ±5% increments. The effects on the overall model outputs of interest were quantified using a sensitivity coefficient [78], defined as:

(6) $$S_{p}^{M}=\frac{\delta M/M}{\delta p/p},$$

where *p* represents the parameter which is varied, *M* represents the system response, and *δM* is the change in *M* due to *δp*, the change in *p*. In the case of the analysis presented here, *M*_1_ corresponds to tumor advance rate and *M*_2_ corresponds to calcification growth rate, while *p* corresponds to any of the parameters varied, as shown in Table III and Fig. 5. In all cases, that larger the absolute change in the sensitivity coefficient, $\left|S_{p}^{M}\right|$, the more sensitive the model is to the given parameter.

We found that the model was most sensitive to changes in cell cycle time ($\tau_{\text{P}}$; duct advance rate |SC| = 2.159 and calcification rate |SC| = 37.42; Fig. 5A, D), while the model was found to be less sensitive to other parameters examined. That is, the magnitude of the percent change in model output (δM/M) was roughly linearly related to change in model input (δp/p); resulting in similar values of SC (i.e., Eq. 6) for the cases examined (maximum values for |SC| in these cases are shown in Table III). These results and the likely causes are examined in greater detail in Discussion.

### D. Necrosis acts as a hypoxia relief mechanism

**Fig. 6.**
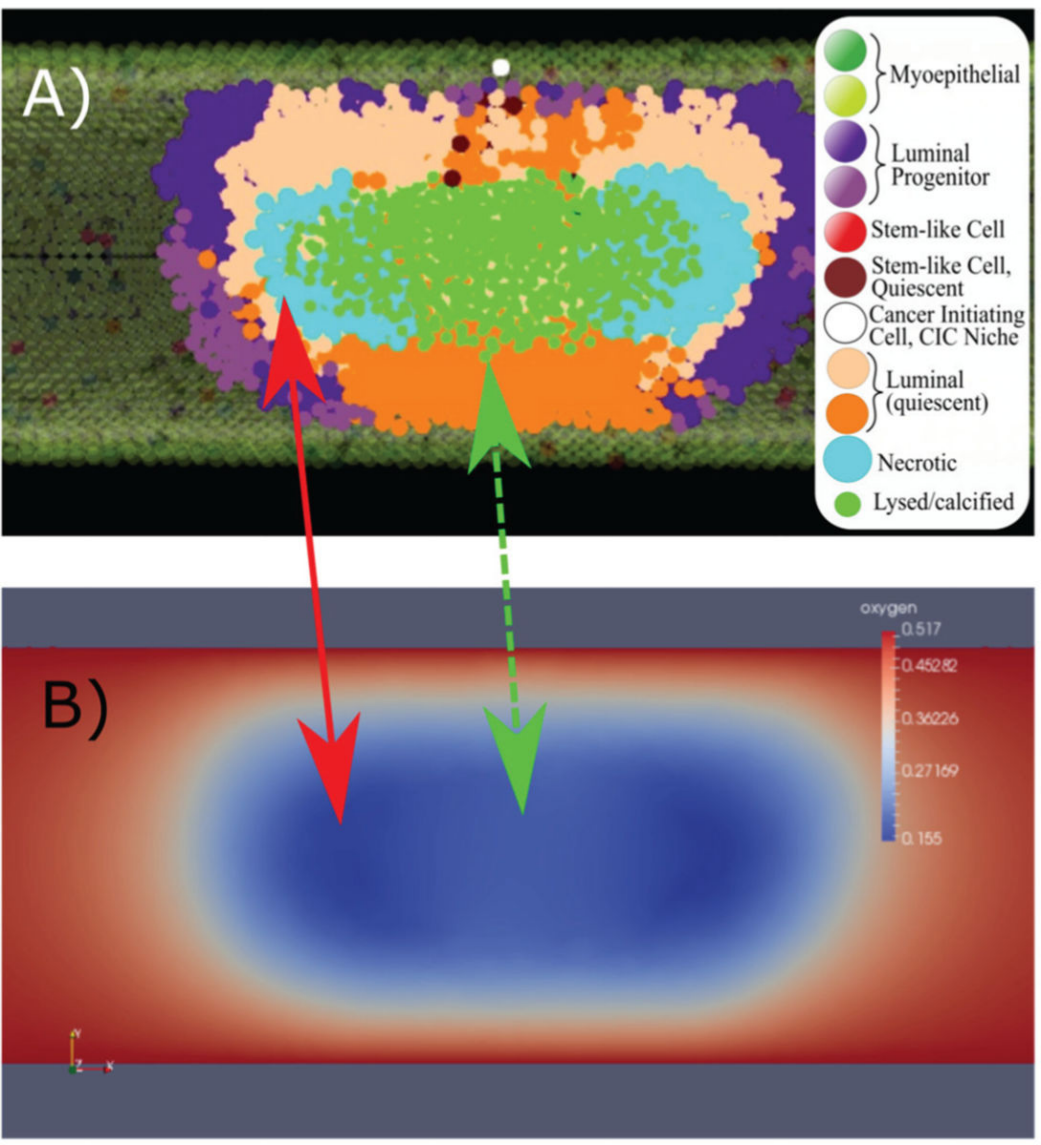
Necrosis acts as a partial relief mechanism for hypoxia. A) Cross sectional view of a section of DCIS with axial necrosis and cell lysis (inset: cell type legend), with the corresponding oxygen solution profile in panel B). Lowest oxygen concentrations were observed in the location of necrotic agents in the DCIS population, following the leading edge of DCIS (panel A, light blue agents and red arrow). In regions of cell lysis and calcification (green agents), oxygen concentrations are observed to raise slightly (green arrow, dashed), due to the relief mechanism of necrosis and calcification through reduction of oxygen consumption at these locations.

Due to the relationship between duct radius and the diffusion distance of oxygen in this tissue (Table I; and also noting that cancer cells consume more oxygen than cells), oxygen remains plentiful in the 100 μm and 150 μm diameter ducts, and hypoxic conditions were only observed in the 200 μm duct; this is consistent with data reported by Mayr *et al.* [71]. In the 200 μm duct, hypoxic conditions were observed to follow the leading edge of the tumor, once the tumor thickness has exceeded the diffusion distance for oxygen radially. Because we assume a constant blood oxygen concentration at the duct boundary, the oxygen threshold at the center of the DCIS mass will remain below the hypoxia threshold (*θH*) unless local cellular oxygen consumption is reduced. While cells may be displaced out of this region, more commonly we observed that this is not the case, due to the high density of cells surrounding this region. Usually, local hypoxic conditions were reduced following hypoxia induced necrosis of agents in the hypoxic region. That is, the death of these cells reduced the oxygen consumption burden in these regions, allowing oxygen concentration to increase slightly in these locations in subsequent time steps, and thus reducing the slope of the oxygen gradient along the duct radius in these locations; this effect is shown in Fig. 6. In this way, necrosis was observed to function as a relief mechanism for hypoxic conditions, allowing local oxygen concentration to rebound slightly, ensuring the remaining cancer population is sufficiently oxygenated, as well as potentially allowing some cells to survive their hypoxia and return to normoxic conditions due to the local oxygen concentration recovery.

### E. Molecular signaling effects

**Fig. 7.**
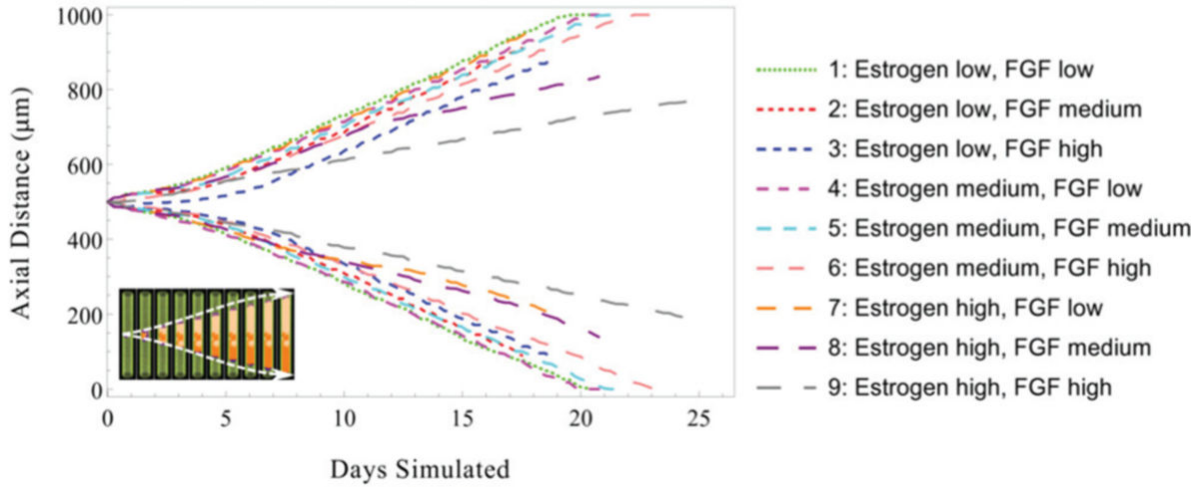
Effects of molecular signaling thresholds on DCIS axial extent. Plot shows the location of the leading edge of the DCIS mass along both axial directions (indicated by upper and lower curve pairs, as shown by white dashed lines in inset, bottom left) under various molecular signaling sensitivity thresholds. Increased molecular signal thresholds for estrogen and FGF resulted in reduced DCIS axial invasion, with high thresholds for both simultaneously showing the most pronounced effect. All results shown are for a 100 μm diameter duct. Case 1 provides a baseline (i.e., with only minimal thresholding effects). Thresholding is implemented using normalized molecular concentrations (1.0 estrogen baseline, with low = 0.8, medium = 0.85 and high = 0.9, and FGF 0.5 baseline low = 0.3, medium = 0.4, and high =0.5). Additional details are shown in Supplemental Materials Fig. S2.

In order to examine the effects of molecular signaling on DCIS, we tested the model in the case of both high and low signaling thresholds for both estrogen (upregulating ER+ cell proliferation) and FGF (upregulating ER− cell proliferation). We define the molecular signaling threshold as the signaling intensity that stimulates a cell to undergo mitosis. Briefly, when thresholds are high, progenitor cells may only be stimulated to mitosis when high local signaling molecule concentrations exceed the high threshold; while low thresholds allow a cells to be stimulated into mitosis under low local molecular concentration. We performed a series of signaling threshold tests to determine the effects of variations in signaling intensity on DCIS progression dynamics in the 100 μm duct. In all tests, we held constant the release and uptake rates for each molecule (i.e., estrogen, AREG, and FGF) across all cells of appropriate phenotype. DCIS axial advance rates were seen to be sensitive to signaling thresholds, as high thresholds limit mitosis, resulting in slower population expansion and fewer DCIS cells over time relative to the low threshold case (Fig. 7, curve 1). Furthermore, this effect was more pronounced in the high estrogen threshold case (Fig. 7, curves 8 and 9), while low estrogen signaling thresholds show lesser reduction in DCIS axial advance, even with high FGF thresholds (Fig. 7, curve 3). This effect is attributed to the upstream to downstream effect of the epithelial to stromal (i.e., estrogen to FGF) signaling pathways, and is examined in additional detail in Supplementary Materials section S2.3 and Fig. S2.

**Fig. 8.**
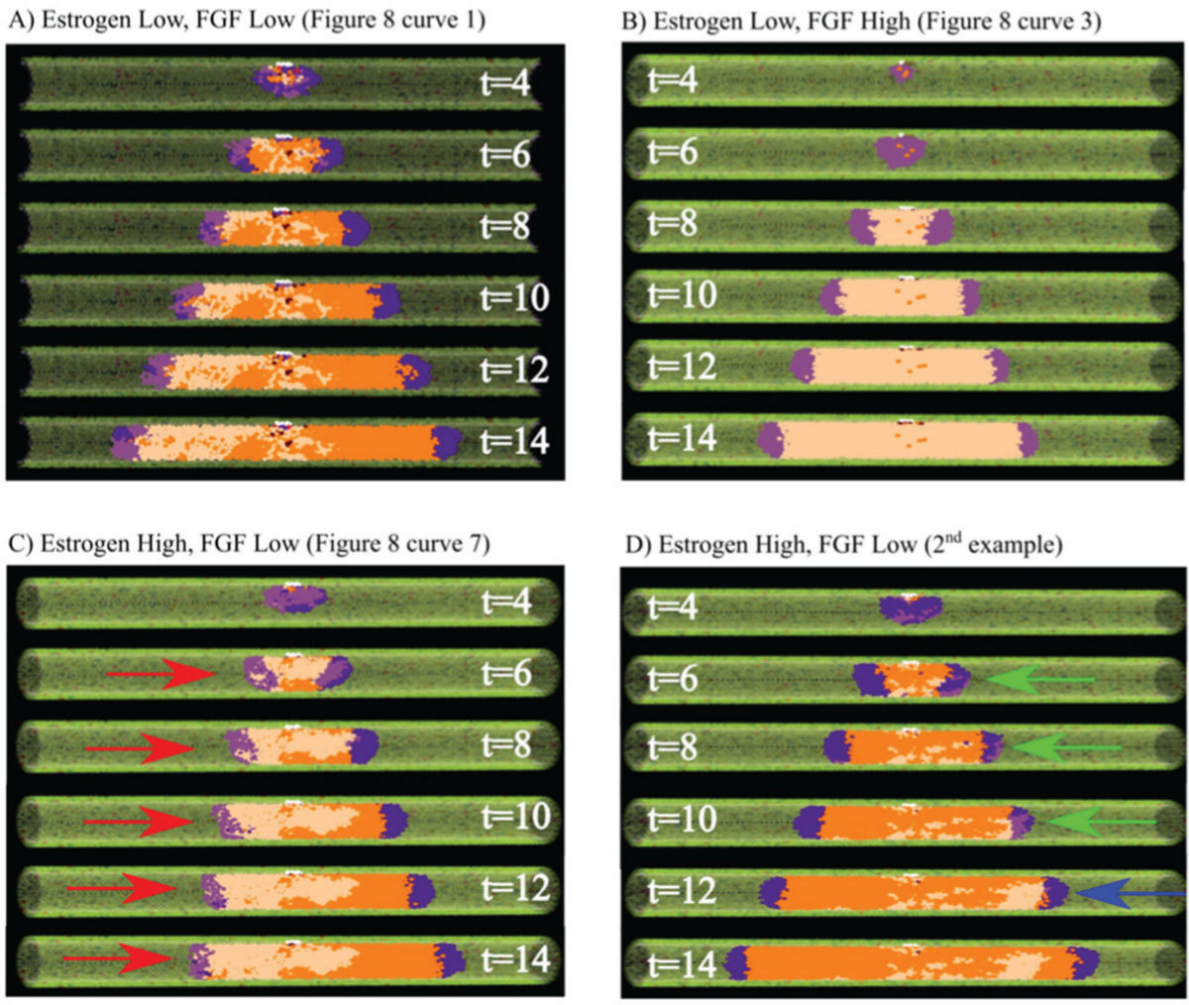
Example simulation results with signaling effects. A) Baseline example without signaling limited proliferation, presented as a cross-section to show the inner phenotype distribution within the DCIS (times shown in days). B) FGF limited proliferation case: the ER− population is completely unable to proliferate at early model times, resulting in the viable rim being completely composed of ER+ cells, resulting in almost the entire DCIS population being an ER+ phenotype. C, D) Estrogen-limited case examples. C) The ER+ viable rim (red arrows) is seen to advance more slowly than the ER− (right side) viable rim due to estrogen limited ER+ proliferation. D) In another simulation instance under the same simulation conditions as C), a mixed viable rim (green arrows at *t* = 6, 8, 10 days) is seen to overcome the proliferation limited ER+ population (due to high estrogen threshold), resulting in an ER+ viable rim (blue arrow, *t* = 12 days) and demonstrating a possible adaptation mechanism to signaling limited cases in the DCIS cancer population. All results shown in a 100 μm duct; cases A–C correspond to curves shown in **Fig. 8** (as indicated), with agent color scheme as shown in Fig. 1C and 7A.

Simulation output from several cases of interest (corresponding to the data shown in Fig. 7) is shown in Fig. 8. An example of a non-threshold limited case is shown in Fig. 8A; it can be seen that the viable rim on the right is completely composed of ER− phenotype, resulting in a large section of completely ER− agents, while the left side is observed to be of mixed phenotype due to the mixed phenotypic distribution within the viable rim. Fig. 8B shows a case of high FGF proliferation thresholds; in this case, the DCIS population is dominated by the ER+ phenotype, likely due to early stage proliferation limitations in the ER− population, resulting in establishment of a completely ER+ viable rim early in the simulation and subsequent development of a completely ER+ tumor. Fig. 8C,D shows two cases of high estrogen thresholding under the same simulated conditions. In the test case shown in Fig. 8C, the ER+ dominated viable rim (left, red arrows) is substantially limited in axial advance rate, in part due to high estrogen uptake in this area (many ER+ cells lower the local estrogen concentration in this region, further reducing mitosis). However, the ER− viable rim (right side) is not limited by the estrogen signaling threshold, resulting in substantially faster axial advance rates. Of note, the ER+ dominated viable rim was not completely arrested in proliferation events, as estrogen may diffuse down the duct (in the direction of the red arrows) from a higher estrogen concentration farther down the duct axis, thereby maintaining some limited proliferation at this location. Fig. 8D shows a case of a mixed phenotype viable rim (green arrows, high estrogen threshold), which allowed the viable rim to adapt to the threshold limitations. The ER− phenotype was able to proliferate more readily than the ER+ phenotype, and overtook it in this case, transitioning the viable rim to only the phenotype not limited by signaling thresholds (ER−, blue arrow). This suggests that the mixture of phenotypes of DCIS may serve as a tumor adaptation mechanism, allowing for tumor progression even when conditions are unfavorable for one or more phenotypes.

## IV. DISCUSSION

We observed that the initial number of TICs at time *t* = 0 only affected DCIS population dynamics at early times (i.e., 2.25−5.9 days). This effect is due to the cell density limitations we impose on the system. At early times after DCIS initiation, cell population increase is exponential, with the number of TICs acting as the base of the exponential. However, once the population expands to reach the cell density-restricted region, cell proliferation is significantly reduced. Subsequent to the exponential growth phase, cell density demonstrated consistent regulation of proliferation events within the TIC niche and in the DCIS population (e.g., see Fig. 4). Under these conditions in our model, DCIS advance through the ducts becomes primarily a function of proliferation events within the leading edge of the tumor, where tumor cell density is the lowest. Because cell density and thickness (i.e., number of cell layers) of the proliferating population at the leading edge is similar in all duct sizes (under the same density conditions in all duct diameters), a similar number of nonquiescent progenitor cell layers was observed in all duct diameters examined, resulting in a consistent linear axial duct advance rate in all duct sizes (but requiring a larger total number of cells in the larger ducts).

Results of model sensitivity analysis showed notable sensitivity to cell cycle time ($\tau_{\text{P}}$), with a shorter simulated cell cycle resulting in faster ductal advance rates (but, importantly, as regulated by estrogen and FGF signaling thresholds; see Table III and Fig. 5). In all cases, larger total DCIS cell populations (due to more total or more frequent cell proliferation events) result in greater duct advance and increased total oxygen consumption (and thus larger hypoxic regions). However, development of hypoxia, necrosis, and calcification is highly dependent on development of a high- density (comedo-like) DCIS completely filling the duct cross- section; when cell density is lower, calcification is reduced, which is due, in part, to local signaling limitations (e.g., Fig. S2E) or development of less dense morphological configurations. Ultimately, calcification is the most downstream process in this model, and is thus the most variable model output, as it is influenced by many other upstream model parameters (Fig. 5E).

Proliferation events within the DCIS population occur at the leading edge of the tumor (where cell density is at the lowest), while hypoxia, necrosis, and calcification occur in the center of DCIS mass (where cell density is the highest). Thus, cell proliferation is not directly decreased by hypoxia and necrosis, as these two phenomena occur in different DCIS subpopulations, and ductal advance rates showed minimal (if any) change due to oxygen and hypoxia thresholds. Further, the effects of FGF signaling variation were also found to be small, demonstrating effective paracrine control over DCIS population dynamics and the ability of phenotypic diversity to overcome signaling limitations. Because FGF production is largely (but indirectly) dependent on the ER+ cell population (through the epithelial to stromal AREG to FGF signaling pathway; see Fig. 1), FGF signaling intensity is directly tied to the ER+ population, which remained fairly constant across all tests, as the estrogen signaling threshold was held at the baseline value for all FGF perturbation tests. Due to this downstream nature of FGF signaling, FGF proliferation restrictions may be overcome by a transition to a predominantly ER+ phenotype in the DCIS leading edge, an example of which is shown in Fig. 8D. Although high FGF thresholds exhibit restrictions on the ER– population, this is balanced by normal (baseline) proliferation in the ER+ population, and only minimal variation was observed in total cell population (and thus axial invasion) in this case.

Our model analysis showed that necrosis functions as a relief mechanism for hypoxic conditions in the tumor, allowing a slight rebound of local oxygen concentration subsequent to necrotic cell death (see Fig. 6). We expect this may play a key role in the natural selection mechanism for a hypoxia-resistant phenotype *in vivo*, where hypoxia-resistant cells are able to outlast the necrotic transition of their hypoxia- susceptible neighbors long enough to benefit from the observed rebound in local oxygen concentration. Interestingly, we observe a predictable, regular distance between the axial extent of calcification and the leading edge of the tumor. This may allow us to make predictions of an effective surgical margin around ducts where calcification is observed with our future modeling efforts (similar to results obtained in 2D by Macklin *et al.*, who were the group first to simulate and examine microcalcifications in DCIS [33]), as the majority of DCIS cases are diagnosed by observation of calcification via mammography [79].

Molecular signaling thresholds functioned as expected, with high thresholds limiting proliferation in the associated phenotype. Of particular interest, as shown in Fig. 8B, the DCIS was seen to be completely composed of the ER+ phenotype in this particular case. Although there are ER− cells near the TIC niche, stochastic proliferation at early times in the simulation run pictured resulted in the viable rim being completely composed of ER+ cells, resulting in an almost completely ER+ phenotype in the DCIS. In this test case, the expanding ER+ population produced AREG, and thus the local FGF concentrations were plentiful to allow for ER– phenotype proliferation at later times in the simulation, but this could not occur as there were no ER– agents in the leading edge. This example demonstrates an important concept: early molecular signing thresholds (or other signaling events early in the DCIS initiation) play an important role in establishing the phenotypes found in the tumor leading edge. If the leading edge is only composed of one phenotype after this period, any future phenotypic diversity in the DCIS population may be due to (or even dependent upon) further mutations within the cancer cell population, or may be derived from de-differentiation events, which are known to occur in mammary cancers (i.e., a cell becomes less differentiated, potentially back to a stem-like phenotype) [80], although at this stage we do not include this phenomena in our model.

In another test case (Fig. 8D), one phenotype becomes dominant when a selective pressure results in reduced proliferation in the other phenotype. In this way, the tumor may adapt to be better suited to survival in its host, likely with ER+ favored in environments with high estrogen signaling, and ER− in cases of higher FGF signaling (or vice-versa in cases of reduced signaling intensity). Because estrogen functions through a system-wide endocrine mechanism, but FGF is a function of the local stroma, these two signaling pathways may be disrupted due to distinct mechanisms in a host, and these may favor one phenotype, analogous to sensitivity mutations in the different cell phenotypes in our model. Although this may potentially serve as a tumor adaptation mechanism, it does so at a cost of sacrificing some phenotypic diversity as currently implemented in our model.

In the future, we will calibrate a set of model parameters (e.g., cell density thresholds and different signaling thresholds) against our own *in vivo* animal data, and will also study the transition from an *in situ* cancer to an invasive cancer. Because our model also includes the surrounding mature duct layer, we will also be able to study the tumor- suppressing effects of p63 in DCIS, which has been shown to come exclusively from the outer myoepithelial layer of the mammary gland [81]. We are also preparing a follow-up study of extensive sensitivity analysis on more parameters using both local and global sensitivity analysis [82–84], as well as investigating how DCIS becomes invasive breast cancer, and how these respond to chemotherapy or other drugs. Ultimately, we will use our model to provide valuable discoveries on the physical and signaling effects that lead to, and are thus indicative of, either consequential or indolent lesions. There is increasing evidence that most DCIS will not become invasive cancer, and women who receive treatment in these cases endure unnecessary pain and anxiety without gaining any benefit [85, 86]. We hope to use our model to gain valuable insights into how overdetection, excessive morbidity, and unnecessary clinical interventions may be reduced, as well as identifying biophysical markers that may be used to distinguish consequential from indolent lesions.

## CONCLUSION

We have presented a 3D, lattice-free hybrid model of DCIS that spans molecular signaling and cell scales in order to examine how phenotypic transitions within the DCIS cell populations due to different signaling events influenced early-time DCIS. Our model has shown agreement with biologically reported data, both in terms of ductal advance rates and extent of calcification [71–73], which we take to be affirmative evidence of its accurate replication of the disease state and predictive power. With the model calibrated as presented, we note that the early exponential growth state of the disease is short-lived, with a rapid transition to a linear growth behavior later on, which is in good agreement with other modeling work [74–77]. In particular, this shows encouraging agreement with early time tumor development rates in existing breast cancer growth models, which have reported logistic or Gompertz growth behavior [87, 88]. Our model showed that a complex interplay of cell density induced quiescence, cell cycle times, molecular signaling, and phenotypic distribution (and likely other factors as well) determines DCIS axial invasion rates and overall disease progression rate. We observed consistent axial invasion rates across all duct diameters tested and consistent distance between calcification extent and the leading edge of the tumor, suggesting full tumor extent and accurate surgical margins may be predictable based on imaging data on a per-patient basis, as is consistent with other DCIS modeling results [33].

## Supplementary Material

supplementalmaterials

## Figures and Tables

**TABLE I:** MODEL PARAMETERS

Model Parameter	Symbol	Baseline Value	Reference
Hypoxia threshold	$\theta_{\text{H}}$	1/3 normoxia	[44, 47]
Oxygen diffusion constant	*D*_oxygen_	2.5 ×10^−6^cms^−1^	[48]
Blood oxygen concentration	$C_{O_{2}}$	100 mmHg	[44]
Estrogen diffusion constant	*D*_estrogen_	2.45 × 10^−6^ cm^2^ s^−1^	*
AREG diffusion constant	*D*_AREG_	3.18 × 10^−7^ cm^2^ s^−1^	[49]**
Proliferation rate (frequency)	$\lambda_{P}$	≤ 1 per 16 hours	[50, 51]
Cell cycle time	$\tau_{\text{P}}$	16 hours	[50, 51]
Progenitor symmetric proliferation probability	$\omega_{\text{P}}$	100%	
Proliferation cycles before differentiation	*P*_max_	50	[52, 53]
Stem cell symmetric proliferation probability	$\omega_{\text{sc}}$	12%	[54]
Mature mammary cell radius	*r*	5μm	[51,55]
Healthy cell oxygen consumption rate	$\lambda_{\text{H}}$	45 attoMol cell^−1^ sec^−1^	[55]
Cancer cell oxygen consumption rate	$\lambda_{\text{C}}$	4.5x healthy cell rate	[55]
Hypoxia time to necrosis	$\tau_{\text{N}}$	12 hours	[56]
Lysis volume increase due to swelling	$V_{\text{L}}$	100%	[57, 58]
Lysis time	$\tau_{\text{L}}$	6 hours	[59]
Time to calcification	$\tau_{\text{C}}$	14 days	[60]
Calcified volume % of pre-lysis cell volume	$V_{\text{C}}$	30%	[61]
Estrogen baseline^†^ (% perturbation)		0.85 (0.05)	
FGF baseline^†^ (% perturbation)		0.5 (0.1)	

Important model parameter baseline values. When not readily available in the literature, diffusion constants were estimated either through interpolation from values from structurally similar molecules (*) or from known values based on relative molecular weights (**). Other uncited values were determined from model calibration.

† Normalized values. We have reduced the value for stem cell symmetric proliferation by 1% from [54].

**TABLE II:** TRANSITION TIMES BETWEEN EXPONENTIAL AND LINEAR RATES OF CELL POPULATION INCREASE

Duct size (μm)	Transition time (days)	Exponentia l fit (*y* = *ae*^*kt*^)	*R* ^2^	Linear fit (*y* = *kt*)	*R* ^2^
100	5.60	14.02*e*^0,714∙*t*^	0.991	251.90 ∙ *t*	0.999
150	7.00	24.93*e*^0,633∙*t*^	0.992	737.09 ∙ *t*	0.998
200	6.38	16.82*e*^0,717∙*t*^	0.992	1164.38 ∙ *t*	0.989

Transition times between exponential and linear cell population increase rates observed across all duct diameters tested. Transition times were determined by iteratively dividing growth data for each duct size tested into exponential and linear subsets; with transition time chosen as the division time that maximized *R*^2^ across both exponential and linear sets. At early times, cell population increase rates (exponential) were observed to be similar across all duct diameters tested, while at later times the linear cell population expansion increased with increasing duct diameter.

**TABLE III:** SENSITIVITY ANALYSIS

Parameter	Days simulated	Tumor advance rate (μm/day)		Calcification growth rate (μm^3^/day)	

		$\left|S_{p}^{M}\right|$	Variation	$\left|S_{p}^{M}\right|$	Variation
Cell cycle time (*τ*_p_)	30	2.159	0.95	37.42	0.95

Hypoxia threshold (*θ*_H_)	30	0.988	0.95	13.30	1.1

Cancer cell oxygen consumption rate (*λ*_C_)	30	0.768	1.05	22.46	1.05

FGF proliferation threshold	30	0.462	1.1	13.82	0.95

Parameters were varied individually as indicated in ±5% intervals (in a range of 0.8- to 1.2-fold of their baseline values), with all other parameters held constant at their baseline values (Table I). All parameters were run for times indicated, with 30 day runs for all cases examined. In each case, the maximum absolute value of sensitivity coefficient $\left|S_{p}^{M}\right|$ gives quantification of overall model sensitivity of the parameter tested, which was observed at the associated reported variation value. The model was found to be most sensitive to variations in the cell cycle time, with lower cell cycle lengths resulting in greater total DCIS cell population and faster axial advance rates.
