# Supplementary material for: A multiscale agent-based model of ductal carcinoma in situ
Source: IEEE Trans Biomed Eng. Author manuscript; Available in PMC 2021 Sep 16. (PMC8445608; doi:10.1109/TBME.2019.2938485)
Supplement: supplementalmaterials [file NIHMS1587309-supplement-supplementalmaterials.pdf]

Supplementary Materials

A multiscale agent-based model of ductal carcinoma in situ

JD Butner, D Fuentes, B Ozpolat, GA Calin, X Zhou, J Lowengrub, V Cristini, Z Wang

SI Text

S1. Continuum methods

S1.1 Parameter quantification for continuum methods

Movement and consumption of oxygen at the cell level is well characterized, with oxygen diffusion constants reported in a wide range, of between 4×10^{-8} to 1.1×10^{-4} cm^2/s [1]; accordingly, we take a somewhat median value of $D_{\text{oxygen}} = 2.57 \times 10^{-6}$ cm^2/s as reported in [2]. Oxygen consumption rate (λ_H) of a single cell is in the 10^{-18}–10^{-16} Mol cell^{-1} s^{-1} range; these are reported in the 2.5–45 $\times 10^{-18}$ Mol cell^{-1} s^{-1} range for healthy cells (lower consumption for mature cells and higher consumption for proliferating cells), with further increased consumption for cancer cells (λ_C) [3]. Specifically, oxygen consumption rates have been measured (in cell culture) up to 150×10^{-18} Mol cell^{-1} s^{-1} for EMTGIRo murine mammary tumor cells in exponential growth (and lower in the plateau phase, at 100×10^{-18} Mol cell^{-1} s^{-1}), and up to 260×10^{-18} Mol cell^{-1} s^{-1} for the MCF-7 breast cancer cell line [3]. In the work presented here, we have taken the upper bound of the reported range for rapidly proliferating cells ($\lambda_H = 45 \times 10^{-18}$ Mol cell^{-1} s^{-1} for full-grown cells), which is adjusted for both cell volume (i.e. is reduced in the case of smaller daughter cells) and cell phenotype (cancer cells consume more oxygen, see **Table 1** main text; λ_C and λ_H, based on [3]). Diffusion constants for AREG, estrogen, and FGF in tissue were less well-characterized, and were thus estimated using linear interpolation from known diffusion coefficients for similar molecules based on relative molecular weights.

S1.2 Phenomenologically determining estimates of model parameters

In the calibration of our model, we encountered several mathematical quantities that we were unable to quantify from literature-reported values. These included diffusion constants for AREG, FGF, estrogen, as well as binding or release rates for these molecules, or the concentrations which upregulate proliferation. To circumvent this issue, we estimated diffusion coefficients for these molecules based on molecules with similar structures and molecular weights, and used normalized concentrations for these molecules within the simulations performed. Specifically, binding and/or release rates (λ_i, equations 3,4) of AREG, estrogen, and FGF were determined using standard parameter estimation techniques (basically, calibrated values which together minimized the discrepancy between simulated and reported DCIS duct advanced rates). Baseline signaling thresholds were estimated under the assumption that the highest literature-reported DCIS invasion rates [4-6] occur under conditions where cell proliferation is uninhibited by unavailability of signaling molecules involved in upregulating proliferation. Testing of the model under circumstances where proliferation was completely uninhibited by any signaling effects revealed that ductal invasion rates of DCIS are capable of exceeding those reported in the literature, and this thresholding plays an important part in regulating cell proliferation and associated duct advance rates. This allowed us to establish a baseline threshold for each molecule where literature-reported invasion rates were observed, and at the same time where all phenotypes were able to continue proliferation within the same simulation. We refer to this baseline signaling threshold as "medium" in **Figures 7** and **S2**.

S1.3 mesh discretization, computational costs, and numerical details

Mesh discretization was on the order of the mature agent diameter, with element length around a median range of 10 μm. While it is not practical to enforce an exact element length across the entire mesh, the simple geometry of the duct domain allowed us to ensure mesh element length was consistent across the entire domain (as there was no need for any localized mesh refinement, etc.). Continuum solutions were obtained via the FEM method using direct linear solvers, 2nd order Gaussian Quadrature, L^2 norm, and 1st order Lagrange (scalar) basis at 60 second intervals, while ABM solutions were advanced in 30-minute intervals. Simulations were executed in serial (single node single core) runs on the Lonestar 5 computer at Texas Advanced Computing Center [7], with all results presented from simulations run for ≤ 48 wall clock hours (except parameter perturbation results in section 3.4, which were run up to 7 days wall clock time).

S2. Discrete methods

S2.1 Cell-cell physics

In our DCIS model, agents are bound to remain within the duct cavity (defined by the luminal inner layer of the mature duct), and may not transition to invasive ductal carcinoma via penetration of the duct basement membrane. Within the duct, agents may move freely as determined by the physics of cell-cell interactions (solved using Bullet Physics, an open-source rigid and soft body physics engine implemented in C++), and all agent coordinates are updated after every cell conformational and position change event (i.e., mitosis, movement, growth, lysis-induced swelling and cell rupture, etc.). Cells may be displaced by events in neighboring agents (e.g., movement, proliferation, growth), and are infinitely compliant to displacement. Subsequent to a cell proliferation or growth event, the movements of the surrounding cells are solved through a series

of dynamically refined (variable) time steps to mechanical relaxation (achieved through both BulletPhysics adaptive internal time steps and through imposition of a fine time step resolution in the physics engine world), allowing the cell physics to return to a new equilibrium configuration before the next proliferation event or advancement to the next simulated time step. In this way, we are able to achieve a high level of stability in the cell-scale discrete model, and avoid any unforeseen behaviors that may result in instability. For example, a large number of simultaneous proliferation events (i.e., all proliferation events scheduled to occur within an ABM time step will be executed at the end of the time step) which is possible taking 30 minute time steps, where all proliferation events that would have occurred within that 30 minutes are executed upon advancement to the next time step) could potentially result in artificially large displacements (and thus large accelerations, which may incorrectly break cell-cell adhesion, etc.) in some cells due to the summation of many smaller simultaneous displacement events. By allowing a return to mechanical equilibrium after each event, we are able to avoid these potential complications and assure reliable stability in the discrete portion of the model without imposing any artificial constraints (for example, this can be accomplished artificially by restricting (clamping) movement to no more than a defined maximum distance per time step).

Cells adhere to neighboring cells through application of cell-cell adhesion forces, and are resistant to displacement due to these forces, as well as static, kinetic, and rolling friction (simulating cell adhesive forces). Cells may also deform due to pressure from surrounding cells, which we represent mathematically as a loss of energy through a coefficient of restitution C_R, represented for each cell i as the square root of the ration of the cell's kinetic energy before and after deformation as $C_{R_i} = \sqrt{\frac{0.5 \cdot m \cdot u_c^2}{0.5 \cdot m \cdot v_c^2}} = \frac{u_c}{v_c}$, where m is the cell mass and v_c and u_c are the cell velocity before and

after cell-cell interaction, respectively (and thus cell deformations are not modeled explicitly as a change in plasma membrane conformation). However, because two cells may not occupy the same space, ultimately cells must be compliant to their neighbors, and thus displacement forces always overcome the forces which resist cell movement, although these forces do play a direct role in determining which displacements will occur.

S2.2 Maximum cell proliferation cycles

Normal mammary epithelial cells have been observed to divide 55–60 times in culture, and even up to more than 250 cycles through immortalization with *c-myc* or its direct transcriptional target, hTERT (the human telomerase subunit responsible for catalysis) [8, 9]. Although cancer cells are known to experience a level of immortalization, we choose to limit the maximum possible proliferation cycles (P_{max}) of the DCIS progenitor phenotype to the lower end of this spectrum, i.e., $P_{max} \leq 50$ mitosis cycles before differentiation (to a terminally differentiated, i.e., non-proliferative state). DCIS progenitors count the number of cycles they have proliferated (akin to a telomere shortening mechanism), and upon hitting the proliferation threshold P_{max} will always give rise to two terminally differentiated daughters. Cells are proliferation restricted by surrounding cell density, and will become quiescent if the local cell density (approximated by true mechanical density, i.e., cell mass/volume within a predetermined distance from each agent) crosses a certain threshold. This restriction is reversible, and if density of the surrounding cells is reduced due to cell death or movement, then a progenitor phenotype cell may resume proliferation. In the model, cells which have proliferated the maximum number of cycles will always give rise to two differentiated daughters in their mitosis cycle. We note that it is possible for P_{max} to exceed the

time simulated in the model runs presented here; in this case, the differentiated phenotype is not observed.

S2.3 Cell density-induced quiescence

We have implemented a somewhat simplified approximation for cell density as a density threshold over a certain number of "cell-lengths" (diameters) around the cell. There are many ways this can be estimated at the discrete cell level; to name a few: a cell in 3D would have contact points with up to 12 neighbors under maximum cell density conditions, mechanical pressure increases with increasing cell density, increased local signaling from increasing cell numbers, etc.. However, the first two are not ideal for this application, as not all cells in high density have 12 contact points at all times due to small fluctuations in cell position, and most mechanical pressure within the *in vivo* DCIS mass likely comes from stretching of the surrounding epithelium and the weight of the surrounding tissue (which we do not include in our model), making true mechanical pressure a poor choice for us as we do not model these. Ultimately, we chose to calculate the actual physical density (mass/volume) locally for each cell.

Taking into account the model simplification that cells are approximated as rigid-body spheres, we may use standard cell lattice sphere packing values (ignoring the fact that the sphere-packing becomes more complicated when spheres are not all equally sized) to obtain a maximum "actual" cell density, which is known to be 0.74 (FCC and/or HCP atomic packing factor) with equally sized spheres. Using this adjustment factor (i.e., that cell 100% cell density occurs at 74%), we may calculate the actual physical density (mass/volume) of cells within a certain number of cell diameters of the cell; that is, its "local density." Cell mass is determined by cell volume (for

simplicity, we assume constant density across all cells), leaving only the question of what is the effective volume over which density should be calculated. That is, now many cell lengths away from the cell of interest does local cell density shown an effect? We estimated this through a calibration method against maximum observed DCIS advance rates measured in vivo; which we assume are representative of a case effectively uninhibited by the signaling pathways we examine. By testing the model over a range of these "effective density distances" under simulation conditions which allowed proliferation to be completely uninhibited by molecular signaling, we were able to determine a value that worked well to consistently replicate measured in-vivo observed DCIS advance rates.

S2.4 Hypoxia, necrosis, and cell lysis

Hypoxia is commonly seen in DCIS in ducts over a certain diameter; hypoxia-induced necrosis has been reported in up to 94% of ducts of diameter > 360 μm, but only 34% of ducts with diameter < 360μm, with an average viable rim thickness of 180 μm before hypoxia onset [4]. This is due, in part, to increased oxygen consumption rates in mammary cancer cells, reported to be 150–260 Mol $cell^{-1}$ s^{-1} for EMTGIRo and MCF-7 cell lines, respectively, relative to 2.5–45 Mol $cell^{-1}$ s^{-1} for healthy cells (depending on cell type and cell phase, i.e., quiescence vs. mitosis phases) [3]; this translates into an increase of up to ~100 fold oxygen consumption in cancer cells. Cancer cells have been reported to enter hypoxia when local oxygen supply drops below 8–10 mmHg [10, 11], and at about 1/3 the normoxia concentration observed in the healthy tissue [10], which is the threshold value we used for the onset of hypoxia in the work presented here (i.e., hypoxia threshold θ_H = 1/3 normoxia).

Cells in the model will enter irreversible hypoxia induced necrosis after a programmed time to necrosis (τ_N = 12 hours of continuous hypoxia) based on values reported in [12]. Subsequent to necrotic death, cells enter a lysis phase, swell under lysis conditions until the plasma membrane ruptures. In the work presented here, time to rupture τ_L = 6 hours as per [13] (mammalian cells may swell from 1−5× up to at least 10× their original volume during lysis [14-16]; we use 2× in this work, as seen in previous DCIS modeling work [17]), resulting in their cytoplasmic contents being released into the luminal cavity. Leaked cytoplasmic contents may then form microcalcifications (as seen in mammographic imaging); here we take the calcified volume (V_c) to be 30% of the volume of the cell at the time of lysis initiation (as cytoplasm is reported to be roughly 70% water [18]). Spilled cytoplasmic contents become calcified after a programmed time to calcification τ_c =14 days (calcification times in 4T1 and 4T1.B cell lines *in vitro* are reported as 11 and 14 days, respectively, with 4T1.B cells more commonly forming microcalcifications *in vivo* [19]; thus we choose 14 days for this study).

S3. Simulation results

S3.1 Number of TIC cells in TIC niche

We assumed that the number of cells undergoing a transition to a TIC phenotype will be minimal, and may occur in one or a small cluster of adjacent cells (as lineage tracing studies of the mature mammary gland homeostasis maintenance process have shown that over time, many contiguous cells may share a common mother, clonal lineage, and genetic makeup [20]). Under this assumption, we tested the effects of initiation of a TIC phenotype in between 1–9 luminal cells

(specifically, 1, 3, 5, 7, and 9 TICs) at $t = 0$ on DCIS population expansion rates and early duct advance rates, as shown in **Figure 4** (main text).

By examining the extent of DCIS advance over time, we discovered that the number of TICs initiated in the niche at time $t = 0$ had a negligible influence on the total DCIS extent and rate of advance for all three duct sizes tested (i.e., 100, 150, and 200 μm in diameter). Average growth rates over the time were estimated by linear fitting of data shown in **Figure 4**, and were found to increase from 11.84 to 11.87 mm/year for the 100 μm duct, 12.82 to 12.89 mm/year for the 150 μm duct, and 10.78 to 11.21 mm/year for the 200 μm duct (a 0.25%, 0.55%, and 3.99% difference, respectively). This result occurs because the disease rapidly transitions from an exponential growth phase to a linear cell-density restricted growth phase, and demonstrates only minimal influence of cell number variations in the TIC niche on duct axial advance rates.

S3.2 DCIS axial advance rates are consistent over all duct sizes tested

To test the influence of duct size on the ductal advance rate of DCIS, we performed simulations in all three duct diameters tested under baseline values (3 simulations for each case), but without the effects of signaling thresholds, as shown in **Figure S1**. Because of the complex interplay between the distribution of cell phenotypes and the associated upstream (e.g., estrogen) and downstream (e.g., AREG, FGF) signaling (also see **Figure 1**, main text), DCIS advance rates under signaling threshold effects may experience significant variation. Thus, we remove this effect to eliminate interaction of these variables to better examine only the effects of variation of the duct diameter. Interestingly, we observed that the variation of DCIS axial advance rates was small between the different duct diameters, with average ducal advance rates (as estimated by linear fitting) averaging

from 11.23 mm/year for the 200 μm duct to 12.95 mm/year for the 150 μm duct (and a more centralized rate of 11.91 mm/year for the 100 μm duct). This relatively small variation of axial advance rate relative to the variation in duct diameter is largely due to the effects of cell density in this case, and is examined more thoroughly in Discussion, main text.

S3.3 Further examination of signaling-limited DCIS invasion

In order to further elucidate the effects of signaling thresholds on DCIS ductal invasion rates, simulations were repeated in triplicate at each of the high, medium, and low thresholds (i.e., **Figure 8**, main text); this is shown in **Figure S2**. It was observed that when thresholds were low (**Figure S2, A-C**), cell proliferation was able to proceed mostly uninhibited due to sufficient endocrine and paracrine molecular concentrations throughout the simulated duct regions. When signaling thresholds were high, proliferation inhibition within the DCIS leading edge due to insufficient signaling stimulation was found to be dependent on the phenotypic makeup of the leading edge. In this case, the ability of the leading edge to continue to advance was dependent on the phenotypic makeup of the leading edge, where presence of the uninhibited phenotype allowed for continued proliferation within that sub-population; this was manifest as slower total axial invasion and larger standard deviation among simulation runs (**Figure S2, G-I**). Moreover, it was observed that restriction in the estrogen signaling caused greater total reduction in axial advance rates, as estrogen acts upstream of the AREG–FGF pathway (this is discussed further in main text). At the medium thresholding level, significant proliferation inhibition was not observed in the simulations performed (**Figure S2, D-F**).

References

1. MacDougall JD, McCabe M. Diffusion coefficient of oxygen through tissues. Nature. 1967;215(5106):1173-4. Epub 1967/09/09. PubMed PMID: 6061810.
2. Sidell BD. Intracellular oxygen diffusion: the roles of myoglobin and lipid at cold body temperature. Journal of Experimental Biology. 1998;201(8):1119-28.
3. Wagner BA, Venkataraman S, Buettner GR. The Rate of Oxygen Utilization by Cells. Free radical biology & medicine. 2011;51(3):700-12. doi: 10.1016/j.freeradbiomed.2011.05.024. PubMed PMID: PMC3147247.
4. Mayr NA, Staples JJ, Robinson RA, Vanmetre JE, Hussey DH. Morphometric studies in intraductal breast carcinoma using computerized image analysis. Cancer. 1991;67(11):2805-12. Epub 1991/06/01. PubMed PMID: 1851048.
5. Thomson JZ, Evans AJ, Pinder SE, Burrell HC, Wilson ARM, Ellis IO. Growth pattern of ductal carcinoma in situ (DCIS): a retrospective analysis based on mammographic findings. British Journal of Cancer. 2001;85(2):225-7. doi: 10.1054/bjoc.2001.1877. PubMed PMID: PMC2364049.
6. Carlson KL, Helvie MA, Roubidoux MA, Kleer CG, Oberman HA, Wilson TE, et al. Relationship between mammographic screening intervals and size and histology of ductal carcinoma in situ. American Journal of Roentgenology. 1999;172(2):313-7. doi: 10.2214/ajr.172.2.9930774.
7. (TACC) TACC. The University of Texas at Austin
8. Wang J, Hannon GJ, Beach DH. Cell biology: Risky immortalization by telomerase. Nature. 2000;405(6788):755-6.
9. Wang J, Xie LY, Allan S, Beach D, Hannon GJ. Myc activates telomerase. Genes & development. 1998;12(12):1769-74. Epub 1998/06/24. PubMed PMID: 9637678; PubMed Central PMCID: PMCPMC316904.
10. Carreau A, El Hafny-Rahbi B, Matejuk A, Grillon C, Kieda C. Why is the partial oxygen pressure of human tissues a crucial parameter? Small molecules and hypoxia. Journal of cellular and molecular medicine. 2011;15(6):1239-53. Epub 2011/01/22. doi: 10.1111/j.1582-4934.2011.01258.x. PubMed PMID: 21251211; PubMed Central PMCID: PMCPMC4373326.
11. Hockel M, Vaupel P. Biological consequences of tumor hypoxia. Seminars in oncology. 2001;28(2 Suppl 8):36-41. Epub 2001/06/08. PubMed PMID: 11395851.
12. Steinbach JP, Wolburg H, Klumpp A, Probst H, Weller M. Hypoxia-induced cell death in human malignant glioma cells: energy deprivation promotes decoupling of mitochondrial cytochrome c release from caspase processing and necrotic cell death. Cell Death Differ. 2003;10(7):823-32.
13. Majno G, Joris I. Apoptosis, oncosis, and necrosis. An overview of cell death. The American journal of pathology. 1995;146(1):3-15. Epub 1995/01/01. PubMed PMID: 7856735; PubMed Central PMCID: PMCPmc1870771.
14. Groulx N, Boudreault F, Orlov SN, Grygorczyk R. Membrane reserves and hypotonic cell swelling. The Journal of membrane biology. 2006;214(1):43-56. Epub 2007/06/29. doi: 10.1007/s00232-006-0080-8. PubMed PMID: 17598067.
15. Wu L-Y, Ma Z-M, Fan X-L, Zhao T, Liu Z-H, Huang X, et al. The anti-necrosis role of hypoxic preconditioning after acute anoxia is mediated by aldose reductase and sorbitol pathway in PC12 cells. Cell Stress & Chaperones. 2010;15(4):387-94. doi: 10.1007/s12192-009-0153-6. PubMed PMID: PMC3082650.

16. Gronroos M, Chen M, Jahnukainen T, Capitanio A, Aizman RI, Celsi G. Methotrexate induces cell swelling and necrosis in renal tubular cells. Pediatric blood & cancer. 2006;46(5):624-9. Epub 2005/07/19. doi: 10.1002/pbc.20471. PubMed PMID: 16025437.
17. Macklin P, Edgerton ME, Thompson AM, Cristini V. Patient-calibrated agent-based modelling of ductal carcinoma in situ (DCIS): from microscopic measurements to macroscopic predictions of clinical progression. Journal of theoretical biology. 2012;301:122-40. Epub 2012/02/22. doi: 10.1016/j.jtbi.2012.02.002. PubMed PMID: 22342935; PubMed Central PMCID: PMCPMC3322268.
18. Luby-Phelps K. Cytoarchitecture and physical properties of cytoplasm: volume, viscosity, diffusion, intracellular surface area. International review of cytology. 2000;192:189-221. Epub 1999/12/20. PubMed PMID: 10553280.
19. Cox RF, Hernandez-Santana A, Ramdass S, McMahon G, Harmey JH, Morgan MP. Microcalcifications in breast cancer: novel insights into the molecular mechanism and functional consequence of mammary mineralisation. British Journal of Cancer. 2012;106(3):525-37. doi: 10.1038/bjc.2011.583. PubMed PMID: PMC3273345.
20. Rios AC, Fu NY, Lindeman GJ, Visvader JE. In situ identification of bipotent stem cells in the mammary gland. Nature. 2014;506(7488):322-7. doi: 10.1038/nature12948. PubMed PMID: 24463516.

Figure S1.

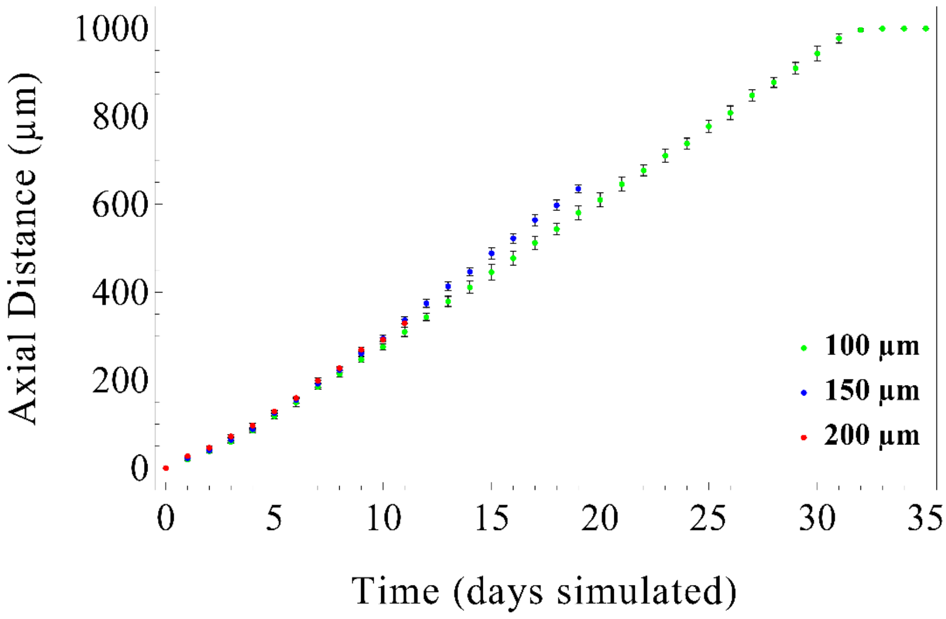

Figure S1. Effect of duct diameter on DCIS axial duct extent over time. Measured DCIS axial extent, corresponding to data shown in **Figure 5** (main text). Axial advance rate and extent of disease is observed to be consistent across all duct axis tested.

Figure S2.

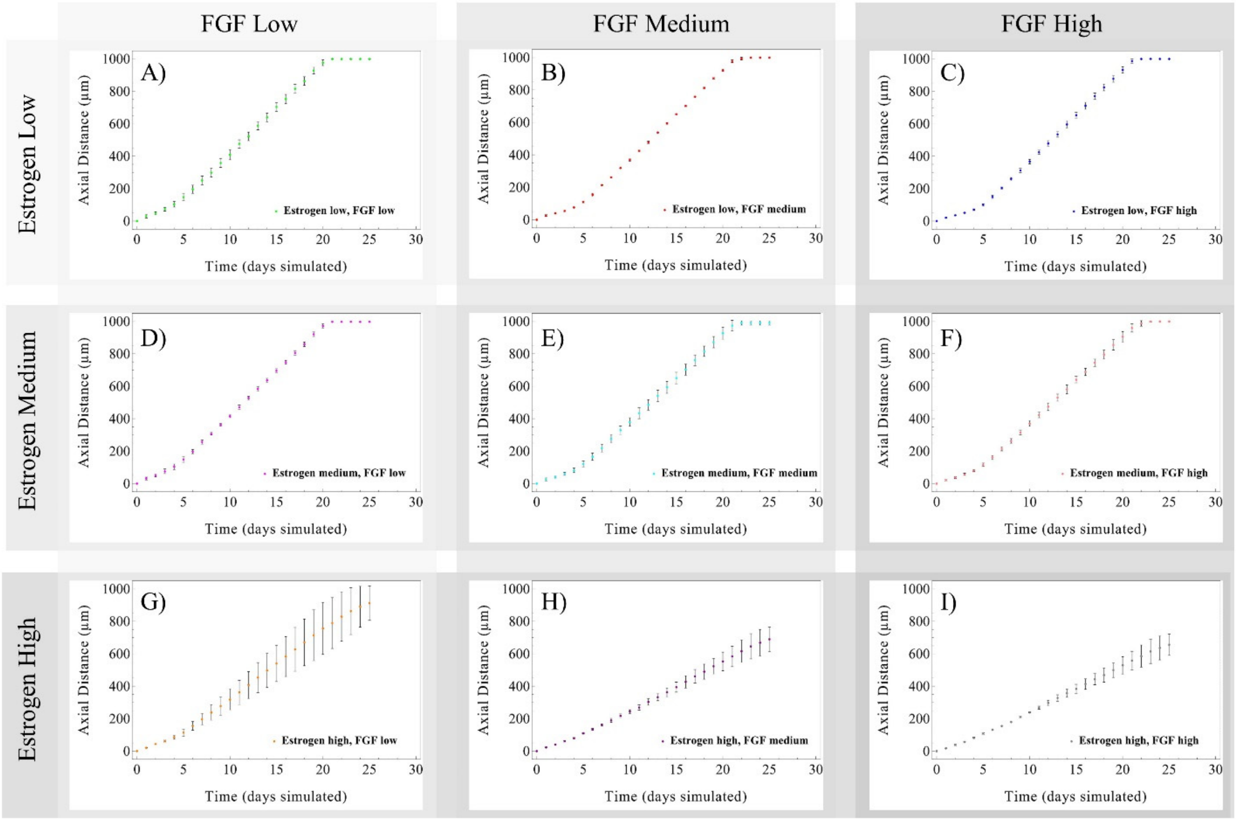

Figure S2: Analysis of effects of signaling thresholds on duct invasion rates. At each indicated estrogen and FGF signaling threshold, simulations were performed in triplicate for 25 days simulated time within a duct section 1000 µm in length and 50 µm in diameter. Each test is shown as mean total axial ductal invasion distance, with standard deviation across all runs indicated by error bars. High thresholds were observed to cause significant inhibition of axial ductal invasion rates, with estrogen-limited profusion having greater effects than FGF-limited diffusion. This is likely due to estrogen acting upstream of FGF, and is explored further in main text.
